# Biomarker evidence for distal tubular damage but cortical sparing in hospitalized diabetic patients with acute kidney injury (AKI) while on SGLT2 inhibitors

**DOI:** 10.1080/0886022X.2020.1801466

**Published:** 2020-08-13

**Authors:** Said Darawshi, Hiba Yaseen, Yuri Gorelik, Caroline Faor, Auryan Szalat, Zaid Abassi, Samuel N. Heyman, Mogher Khamaisi

**Affiliations:** a Department of Medicine D, Ruth & Bruce Rappaport Faculty of Medicine, Rambam Health Care Campus, Technion-IIT, Haifa, Israel; b Clinical Research Institute, Rambam Health Care Campus, Haifa, Israel; c Department of Medicine, Hadassah Hebrew University Hospital, Jerusalem, Israel; d Department of Physiology, Ruth & Bruce Rappaport Faculty of Medicine, Technion-IIT, Haifa, Israel; e Department of Laboratory Medicine, Rambam Health Care Campus, Haifa, Israel

**Keywords:** Acute kidney failure, SGLT2 inhibitors, hypoxia, biomarker, NGAL, KIM-1

## Abstract

**Background:**

Inhibitors of sodium-glucose co-transporter-2 (SGLT2i) were found to improve renal outcome in diabetic patients in large prospective randomized trials. Yet, SGLT2i may acutely reduce kidney function through volume depletion, altered glomerular hemodynamics or intensified medullary hypoxia leading to acute tubular injury (ATI). The aim or this study was to prospectively assess the pathophysiology of acute kidney injury (AKI) in patients hospitalized while on SGLT2i, differing ATI from pre-renal causes using renal biomarkers.

**Methods:**

Serum and urine Neutrophil Gelatinase-Associated Lipocalin (NGAL) and Kidney Ischemia Molecule (KIM)-1, markers of distal and proximal tubular injury, respectively, were determined in 46 diabetic patients who were on SGLT2i upon hospitalization with an acute illness.

**Results:**

Serum and urine NGAL, but not KIM-1, were significantly increased in 21 of the patients who presented with AKI upon admission, as compared with 25 patients that maintained kidney function. Both serum and urinary NGAL correlated with the degree of impaired renal function, which in many cases was likely the result of additional acute renal perturbations, such as sepsis.

**Conclusions:**

Increased urinary and serum NGAL indicates that ATI, principally affecting distal tubular segments, may develop in some of the patients hospitalized with an acute illness and AKI while on SGLT2i. It is suggested that intensified medullary hypoxia by SGLT2i might be detrimental in this injury. By contrast, concomitantly unaltered KIM-1 might reflect improved cortical oxygenation by SGLT2i, and may explain an overall reduced risk of AKI with SGLT1i in large series. The independent potential of SGLT2i to inflict medullary hypoxic damage should be explored further.

## Introduction

Diabetic nephropathy, the leading cause of chronic kidney disease (CKD) in developed countries, is associated with enhanced morbidity and mortality and with substantially increasing medical expenses [1]. The introduction of sodium-glucose-cotransporter 2 inhibitors (SGLT2i) as a novel treatment option for non-insulin-dependent diabetes (NIDDM) revolutionized the management of NIDDM, as it substantially improved overall and cardiovascular outcome among high-risk patients [2–4]. Furthermore, the progression of CKD in patients with diabetic nephropathy has slowed down. These advantages turned SGLT2i into a preferential treatment option among NIDDM patients [5,6]. The renoprotective properties of SGLT2i are thought to be related to the reversal of excessive afferent arteriolar vasodilation, with restoration of glomerular hemodynamics. Indeed, a modest initial decline in glomerular filtration rate (GFR) is an inherent property of SGLT2i, as it reduces trans-glomerular pressure gradient. Nevertheless, this minor effect is reversible, and as with angiotensin-converting-enzyme inhibitors (ACE-i) and angiotensin-receptor blockers (ARBs) [7,8], it provides renal protection on the long-run with an attenuation of the accelerated decline in GFR, characteristic of diabetic nephropathy.

SGLT2i also reduce the incidence of acute kidney injury (AKI), as illustrated in large-scale phase III studies with Empaglifluzin, Dapaglifluzin and Canaglifluzin [2–4], underscoring the renal safety of SGLT2i. SGLT2i were not found to increase the incidence of AKI also in real-life primary care settings [9]. However, case series and FDA registries of AKI in patients using SGLT2i do raise concerns [10,11]. The reason for the discrepancies between such real-life reports and the outcomes in the large prospective randomized series is yet to be defined. Nevertheless, few plausible and physiologically reasonable causative mechanisms have been proposed in cases of AKI presumably linked to SGLT2i. Some patients may have a more prominent decline in trans-glomerular pressure, in particular with the concomitant treatment with ACEi and ARBs. Furthermore, the natriuretic and diuretic properties of SGLT2i can lead to a more pronounced decline in kidney function due to effective volume depletion and dehydration. Caution has, therefore, been called regarding proper monitoring of the hydration status and blood pressure and treatment modifications as needed in patients with concomitant diuretic treatment, renin-angiotensin blockade or with acute medical conditions that may lead to dehydration, such as diarrhea. Additionally, concern has been raised regarding hypoxic tubular injury in the renal outer medulla, resulting from displacement of tubular sodium transport from proximal- to distal nephron segments [12].

The current study was aimed to identify acute tubular injury (ATI) in patients with AKI while treated with SGLT2i, and to differentiate these patients from those with pre-renal failure, induced by effective volume depletion or altered glomerular hemodynamics. Unfortunately, clinical and laboratory parameters that help differing pre-renal failure from ATI might be of little value in diabetic patients on SGLT2i, as orthostatic hypotension can represent autonomic nephropathy, and renal sodium handling and urine concentration might be affected by SGLT2i and other medications. Kidney biopsy is seldom performed in such scenario, and is of limited clinical value principally due to non-timely and non-diagnostic minute tissue sampling [13,14]. On the other hand, biomarkers of tubular injury, detected in serum or urine samples could be helpful, and being nephron-segment-specific, they may provide insight regarding the distribution pattern of tubular injury [14,15]. Herein we report increased plasma levels and urine excretion of neutrophil gelatinase-associated lipocalin (NGAL), a biomarker of distal tubular segments, among hospitalized patients on SGLT2i presented with AKI, but unaltered urine and plasma levels of Kidney ischemia Molecule (KIM)-1, a biomarker originated from proximal tubules.

## Methods

This prospective single-center study was conducted at Rambam Health Care Campus in Haifa, Israel between July 2017 and September 2019. The study was approved by the Institutional Review Board, in accordance with NIH guidelines (IRB-0190-17). Medical records of 6990 newly admitted patients to the Division of Medicine were screened daily (with the exception of weekends) for type 2 diabetic patients treated with Empagliflozin or Dapagliflozin, the two currently available SGLT2i in Israel. Included in the study were patients aged >18 admitted with an acute illness with documented baseline kidney function, who were on SGLT2i upon admission and agreed to participate in the study.

Baseline creatinine of these patients in the community was checked and compared with creatinine levels upon admission and within 24 h from admission. Patients were classified as those with or without AKI, based on the AKI definition according to the 2012 Kidney Disease Improving Global Outcomes (KDIGO) Clinical Practice Guideline for Acute Kidney Injury (AKI). In brief, AKI was defined as an increase of 0.3 mg/dL in blood creatinine levels on admission or within 24 h of hospitalization in two repetitive samples, compared to the highest creatinine levels during the preceding year, and/or an increase in blood creatinine levels of more than 50% of baseline creatinine determined in the 7 days preceding hospitalization. Estimated glomerular filtration rate (eGFR) was calculated using the CKD EPI equation. Following receiving patients' approval and informed consent, blood and urine samples were collected for renal biomarkers within 24 h from admission and kept at −20^°^C until their determination, conducted simultaneously for the entire cohort. Hemoglobin A1c (HbA1_C_), serum creatinine, blood urea nitrogen (BUN), serum electrolytes, urine creatinine and urine electrolytes were determined in the hospital's routine biochemistry laboratory. The medical records of the enrolled patients were retrieved and analyzed for diagnoses, co-morbidities and clinical outcome. Of note, patients previously diagnosed with CKD were defined as such, with stages categorized according to baseline eGFR. Vital signs, blood gases, chemistry, and complete blood count obtained on admission were used for data assessment, as were follow-up creatinine values during the hospitalization course. Noteworthy, SGLT2i administration was held in all patients upon admission but resumed in most on discharge, whenever the principal causes of AKI were detected and managed.

### Determination of KIM-1 and NGAL levels

Serum and urinary KIM-1 and NGAL levels were measured in stored samples (−20^°^C) with commercially available enzyme-linked immunosorbent assay kits as described previously [16], obtained from Wuhan EIAab Science, Wuhan, China (kit number = E0785) and from Abcam, Cambridge, MA, USA (kit number = AB113326), respectively. Urine biomarker levels were normalized for urine creatinine.

The NGAL kit employs a monoclonal antibody specific for human Lipocalin 2 monomer coated on a 96- well plate. The kit for KIM-1 is ultrasensitive, reliably measuring as little as 1.2 pg/ml of KIM-1. The kit uses a monoclonal antibody to KIM-1 immobilized on a microtiter plate to bind the KIM-1 in the standards or sample.

### Statistical evaluation

Data analysis was carried out with the Crunch statistical software (Oakland, CA). Values are presented in the text and table as means ± SD, and in figures as means ± SEM. Student-*t*-test, Chi square likelihood ratio and Pearson's correlations were used as indicated and statistical significance was set at *p* ≤ .05.

## Results

Out of 250 detected diabetic adult patients hospitalized with an acute illness while on SGLT2i, 46 individuals were included in the study, with all withdrawals related to declining participation or to unavailable basal kidney function. Table 1 illustrates the clinical characteristics of 21 of these patients who developed AKI upon admission, as compared with additional 25 patients who did not present with AKI. Most patients (88%) were treated with Empagliflozine and the remaining were on Dapagliflozine. The two groups of patients had statistically comparable age, gender distribution and duration of SGLT2i treatment. The groups were furthermore similar regarding HbA1c levels, the extent of obesity, dyslipidemia and micro- and macrovascular complications. Importantly, baseline creatinine values and the distribution of CKD severity were comparable as well. Yet, basal eGFR was slightly lower in patients who developed AKI. The two groups were also equal regarding co-morbidities and medications proposed as predisposing to SGLT2i-related AKI. By contrast, significantly more patients in the AKI group presented on admission with infection or dehydration/volume depletion, but not with decompensated heart failure. Soft-tissue infections and diabetic foot-associated osteomyelitis were the commonest identified sources of infection (70%), whereas, surprisingly only two patients presented with urinary tract infection, one of them with concomitant osteomyelitis. Volume depletion has been attributed to the use of diuretics (furosemide with or without spironolactone) in all but one of the dehydrated patients.

**Table 1.** Demographic and clinical data at baseline and upon admission.

	AKI Group (*n* = 21)	No AKI Group (*n* = 25)	*p* Value^a^
Age (years)	67.7** **±** **9.9	65.7** **±** **7.8	NS
Male Gender	66%	72%	NS
SGLT2i : Empagliflozine /Dapagliflozine	81% / 19%	76% / 24%	NS
Duration of treatment (months)	10.3 ± 4.7	8.2 ± 5.6	NS
Co-morbidities: (%)			
Obesity	76%	68%	NS
Hypertension	100%	96%	NS
Dyslipidemia	100%	96%	NS
Microvascular disease^a^	62%	60%	NS
Macrovascular disease^b^	90%	68%	NS
Active smoker	24%	24%	NS
Plausible predisposing Medications (%):			
Diuretics	10%	8%	NS
RAAS blockade	38%	48%	NS
Diuretics + RAAS block	43%	24%	NS
NSAID	5%	0 %	NS
Diagnosed illness predisposing to AKI (%):			
Infection	43%	8%	.025
Dehydration / volume depletion	24%	0%	.007
Heart failure	29%	20%	NS
Blood pressure (mmHg)^d^	137 ± 22/69 ± 14	134 ± 23/75 ± 13	NS
Heart rate (beats per minute)^d^	83 ± 19	78 ± 16	NS
HbA_1_C (%)^c^	8.9 ± 2.1	8.1 ± 1.5	NS
Baseline Creatinine (mg/dl)^c^	1.03 ± 0.2	0.97 ± 0.31	NS
Baseline eGFR (mL/min/1.73 m2)^c^	69 ± 13	78 ± 19	.09 (NS)
CKD stage: 1 / 2 / 3a / 3b (%)^c^	20/48/27/5	28/40/16/4	NS
Serum Creatinine (mg/dl)^d^	1.82 ± 0.60	1.08 ± 0.31	.002
Serum BUN (mg/dl)^d^	41 ± 22	22 ± 11	.0004
Fractional sodium excretion (FeNa, %)^d^	0.88 ± 0.70	1.35 ± 1.50	NS
Urine/plasma creatinine ratio^d^	40.3 ± 36.2	62.4 ± 47.5	.08 (NS)
Serum urea/creatinine ratio^d^	22 ± 13	20 ± 8	NS

Values are presented as means** **±** **SD, or as percentage. *p* Values are for student – t-test or for likelihood ratio chi square analysis (for non-parametric values).

^a^ Non-renal microvascular disease: retinopathy, neuropathy.

^b^ Macrovascular disease: ischemic heart disease or stroke and peripheral vascular disease.

^c^ Baseline pre-hospitalization values, The CKD-EPI Creatinine Equation.

^d^ On admission.

All patients in these series survived the hospitalization and none required renal replacement therapy. Kidney function improved in all AKI patients, with 62% of them reaching pre-hospitalization values.

**Figure 1.**
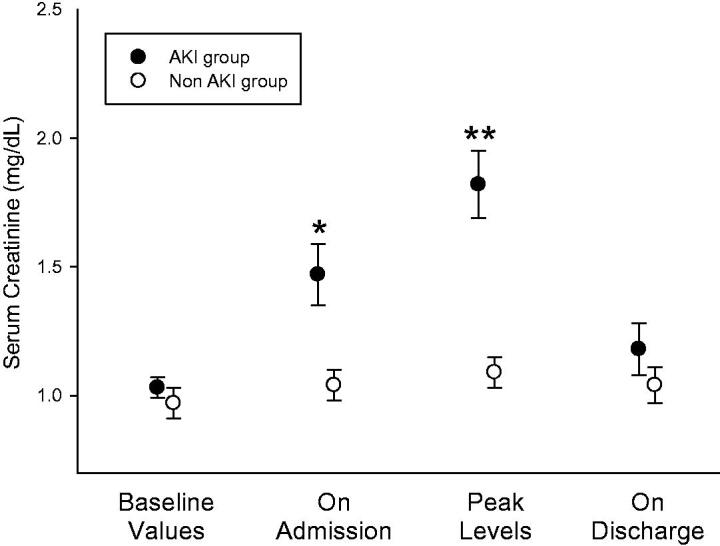
Serum creatinine in the AKI and non-AKI groups. While baseline (pre-hospital) levels are comparable, serum creatinine is significantly higher upon admission (* *p* = .002) and at peak levels during hospitalization (** *p* < .0001, means ± SEM, student *t*-test).

**Figure 2.**
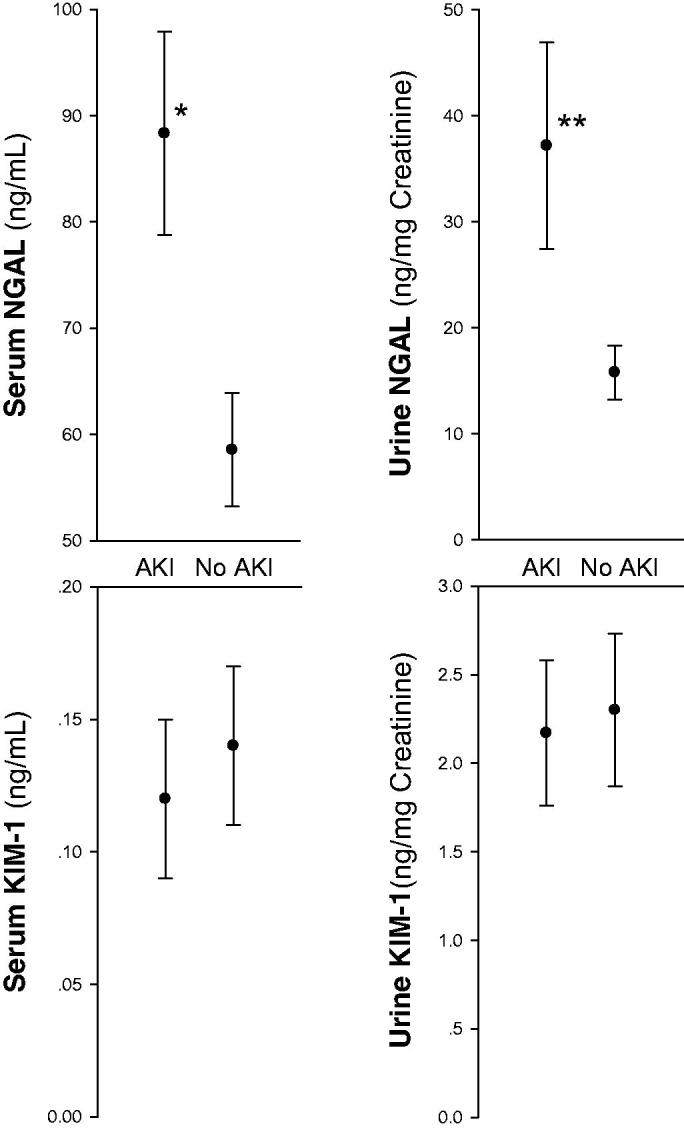
Serum and urine NGAL and KIM-1 in the AKI and non-AKI groups (*n* = 21 and 25, respectively). While KIM-1 levels are comparable in the two groups, NGAL levels both in serum and urine samples are significantly higher in the AKI group (*p = .006 and ** *p* = .032, respectively, means ± SEM, student *t*-test).

**Figure 3.**
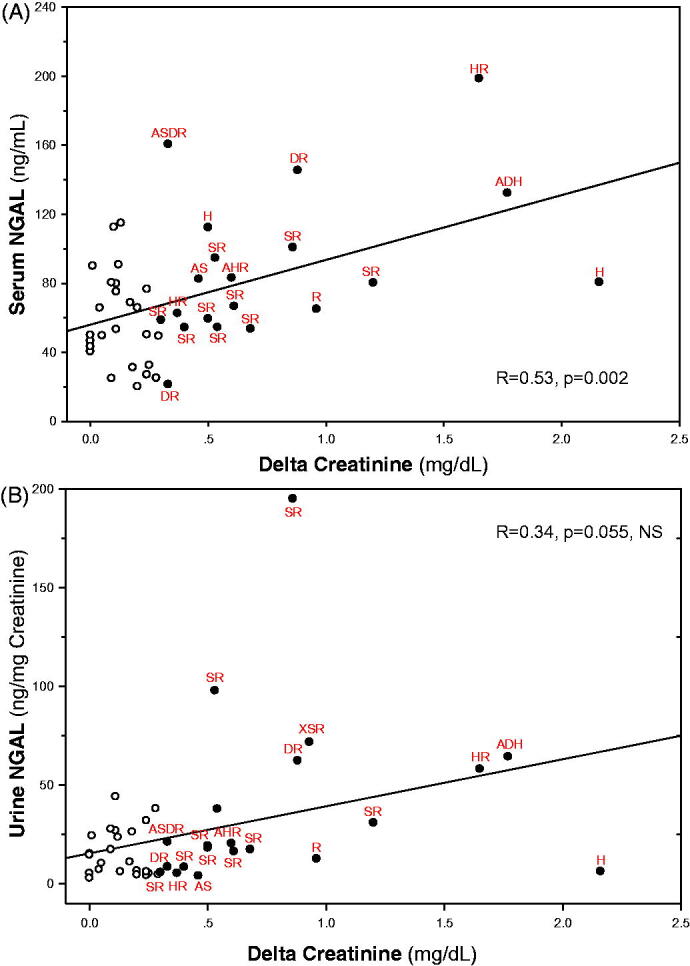
Correlations between serum or urine NGAL and acute renal dysfunction. In (A) serum NGAL levels correlates with maximal rise in serum creatinine from baseline pre-hospitalization levels (delta creatinine, *R* = 0.53, *p* = .002). The correlation between urine NGAL and delta creatinine (B) falls short of statistical significance (*R* = 0.34, *p* = .055, NS, Pearson's correlations for the entire cohort of patients). Patients with AKI are presented as filled symbols, with their potential contributing factors underscored by adjoining descriptions. S: sepsis/infection; H: heart failure; D: dehydration/volume depletion; R: RAAS blockade; A: age ≥ 75, X: NSAID other than aspirin.

Figure 1 illustrates plasma creatinine in the two groups at baseline (pre-hospitalization values), upon admission, at peak levels and upon discharge. All peak levels were recorded within 48 h from admission. NGAL and KIM-1 levels, determined within 24 h from admission, are illustrated in Figure 2. NGAL serum (sNGAL) and urine levels, normalized to urine creatinine (uNGAL), were significantly higher in the AKI group as compared with the control group without AKI (88.32 ± 32.00 ng/ml vs. 58.54 ± 26.65 ng/ml, *p* = .006, and 37.18 ± 44.78 ng/mg creatinine vs. 15.79 ± 12.19 ng/mg creatinine, *p* = .032, respectively). In contrast, serum and urine KIM-1 values (sNGAL, uNGAL) did not rise and were in fact even slightly lower in the AKI group. Interestingly, as shown in Figure 3, the decline in kidney function, determined as delta-creatinine from baseline to peak levels, correlated with sNGAL (*R* = 0.53, *p* = .002), with a near-significant association with uNGAL (*R* = 0.34, *p* = .055, NS). Of note, these figures likely illustrates a mixture of AKI mechanisms, with some patients (at the left-lower part and below the regression lines) conceivably representing pre-renal failure without elevated NGAL levels, others with elevated biomarker, plausibly reflecting tubular injury, and likely patients with mixed renal and pre-renal components. In that perspective, the figures demonstrates the limited diagnostic value for the diagnosis of ATI and its distinction from pre-renal failure in patients with AKI using a single spot test of the biomarker.

As with delta-creatinine, peak creatinine significantly correlated with sNGAL (*R* = 0.49, *p* = .005) and with uNGAL (*R* = 0.40, *p* = .024). uNGAL and sNGAL were also directly inter-correlated (*R* = 0.41, *p* < .007).

## Discussion

AKI related to SGLT2i may range from pre-renal failure, caused by either effective volume depletion or altered glomerular hemodynamics, to true ATI, likely related to hypoxic damage [12]. The major finding reported here is that sNGAL and uNGAL, biomarkers of distal tubular damage [17], significantly increased in hospitalized patients treated with SGLT2i who presented with AKI upon admission, as compared with hospitalized patients treated with SGLT2i who did not develop AKI. Furthermore, NGAL values both in the serum and urine significantly correlated with the magnitude of acute renal functional impairment. These findings suggest that ATI affecting medullary tubular segments was present in many of these patients. By contrast, sKIM-1 and uKIM-1, generated in proximal tubular segments, were not increased in AKI patients as compared to the control group without renal functional impairment, implying the absence of proximal tubular injury, despite renal functional derangement.

The outer medulla is physiologically hypoxic, the consequence of limited regional blood and oxygen supply, combined with intense oxygen consumption for tubular transport, carried out chiefly by medullary thick ascending limbs (mTALs) [18]. In anesthetized rats SGLT inhibition decreases proximal tubular transport, leading to improved cortical pO_2_. By contrast, medullary physiologic hypoxia intensifies, likely due to increased solute delivery to distal nephron segments, enhancing medullary tubular transport work and oxygen consumption [19]. Indeed, Dapagliflozin enhanced Hypoxia-Inducible Factor (HIF) expression in mice kidneys, reflecting reduced tissue oxygenation [20]. In humans, SGLT2i increased plasma erythropoietin and induced reticulocytosis [21], likely reflecting intensified hypoxia at the corticomedullary junction [22]. Thus, it is tempting to assume that while hypoxic injury might be mitigated in cortical tissues in patients treated with SGLT2i, hypoxic medullary injury may develop, particularly when mechanisms that match regional blood flow and tubular transport are hampered, or in the presence of additional conditions that alter medullary oxygenation (such as CKD, diabetes or aging) [18,23]. Indeed, the diabetic kidney is especially prone to hypoxic medullary injury, as outlined in detail elsewhere [24]. This has led us to propose the avoidance of concomitant use of NSAIDs and to discontinue SGLT2i before the administration of iodine-based radiocontrast agents [25], since these interventions are characterized by significant intensification of medullary hypoxia [26].

The use of biomarkers of AKI in the detection and assessment of AKI is expanding, and combined assays of several biomarkers enhance their sensitivity and specificity [14,27,28]. Yet, while their rise in young and stable patients with intact kidneys is highly indicative of acute renal injury [29,30], they are less predictive in older patients with additional morbidities, particularly preexisting renal impairment [14]. Therefore we used as a control group a comparable cohort of hospitalized adult diabetic patients on SGLT2i treatment upon admission who did not develop AKI. Despite the small numbers of patients, we could demonstrate higher plasma and urine concentration of NGAL among those who developed AKI, as compared with the control group. The rather selective rise in NGAL but not KIM-1 suggests the presence of medullary hypoxic damage, in line with our expectations, based on the evidence for SGLT2i-induced medullary hypoxia [12,19], as the former marker is principally generated in the kidney by mTALs and collecting ducts, whereas KIM-1 is expressed in proximal tubules [15]. The unchanged sKIM-1 and uKIM-1 further excludes a nonspecific tubular injury unrelated to renal oxygenation gradients and medullary hypoxia. One may even speculate that the unaffected and even slightly lower sKIM-1 and uKIM-1 in the AKI group could reflect cortical tubular protection, related to improved cortical oxygenation by SGLT2i [19]. This may explain the overall safety of SGLT2i regarding the incidence of AKI in large prospective studies, with hypoxic medullary ATI seldom occurring at the presence of acute or chronic predisposing factors that alter medullary oxygen balance.

The two groups of patients were comparable regarding co-morbidities and the use of medications that could predispose to pre-renal AKI. Yet, among acute medical conditions, significantly more patients with AKI presented with infection or dehydration/effective volume depletion, determined on clinical grounds and assessed as causing renal functional impairment. These acute conditions alone could have contributed to AKI. Yet, conversion from pre-renal azotemia to ATI takes time [31]. Furthermore, sepsis-induced AKI is believed to chiefly represent altered glomerular hemodynamics with efferent arteriolar vasodilation [32], while tubular injury, if present, is rare and focal, involving cortex as well [33]. Thus, with biomarker-evidenced medullary but not cortical tubular injury, it is tempting to assume that SGLT2i may play in concert with such acute renal insults, adding a component of medullary hypoxic stress.

There was a trend to a lower eGFR at baseline in the AKI group, suggesting CKD with diminished renal reserve, a known risk factor for hypoxic AKI [34]. Age is also an acknowledged risk factor for hypoxic AKI, likely reflecting reduced renal reserve and the inability of the aged kidney to improve medullary oxygenation [35]. Though our experimental groups are comparable regarding mean age, 5 patients in the AKI group were 75 years or older, as compared with 1 patient, only, in the non-AKI group. Though this difference falls short of statistical significance, it is in line with our concept of the role of medullary hypoxia and with the recently reported trend of increased rate of dehydration and AKI among patients in this age group, noted by sub-group evaluation of the DECLAIR safety analysis [36].

Among patients on diuretics on admission, 8 (38%) in the AKI group were treated with loop diuretics as compared with 6 only (24%) in the non-AKI group. Though this difference in not statistically significant, this finding is somewhat unexpected. Loop diuretics improve medullary oxygenation by inhibiting tubular transport in medullary thick limbs [37]. Indeed, these agents prevent medullary hypoxic damage [38] and improve HIF-mediated hypoxia response [39]. Therefore, one could anticipate an inverse trend for ATI, namely that NGAL, reflecting medullary hypoxic damage, should be lower in AKI patients on loop diuretics. Yet, dehydration was likely intensified with loop diuretics. Furthermore, with the short elimination half-life of loop diuretics, often given once or twice a day, active transport in mTALs is enhanced most of the time where the effect of the drug weans off [40].

Our findings illustrate the limited value of commonly used indices that help differing between pre-renal AKI and ATI, such as fractional sodium excretion, urine/plasma creatinine ratio, or plasma BUN/creatinine ratio, as these indices were statistically comparable in the two studied groups. These parameters and likely additional tests such as urine osmolality are especially useless in patients on SGLT2i, with disrupted sodium handling and urinary concentration. The regression lines illustrated in Figure 3 also exemplify the limited specificity and sensitivity of biomarker spot-tests, with many patients classified as AKI according to KDIGO criteria showing biomarker levels comparable to patients without AKI. Excellent predictive values where achieved in prospective studies were biomarkers were taken serially before- and following an insult [29,30]. Yet, despite an overall statistically significant difference in NGAL for the entire cohort, the marked overlap in biomarker levels, questions the diagnostic value of such parameters in the evaluation of individual patients. The overlap in NGAL in serum and urine among patients with or without AKI possibly reflects untimely sampling concerning the injury process, or more likely, the mixed mechanisms of renal functional impairment associated with diverse clinical conditions, including SGLT2i administration, with predominant pre-renal factors in some cases, hypoxic injury in others, or their mixture. The overlapping uNGAL in the two groups of patients is especially prominent in Figure 3(B), where ‘normal’ urine NGAL levels are more clustered. uNGAL physiologically should better predict distal tubular injury since its majority comes from the distal nephron, whereas sNGAL also reflects diminished renal clearance of NGAL originated in non-renal tissues [17,41]. Unfortunately, urinalysis, a most important diagnostic tool for the detection of ATI [42] has not been utilized in most patients, and its correlation with the biomarkers could not be tested.

To our knowledge, this is the first report prospectively assessing AKI biomarkers in hospitalized patients admitted while on SGLT2i treatment. Noteworthy, however, our study has substantial limitations. The number of patients is small, which precludes a proper compensatory matching, and may lead to statistical errors related to small sample size. Urinalysis is not provided and likely, the use of NSAIDs has been under-reported. The value of plasma creatinine for the assessment of glomerular filtration in patients with unsteady kidney function is limited. Furthermore, though we used a kit specific for NGAL monomers, released from distal tubular segments, since NGAL dimmers originate from neutrophils [43], our findings indicating a rise in sNGAL and uNGAL could be affected by infection, occurring more frequently in the AKI group [44]. Most importantly, since the majority of patients presented with an acute illness, some with organ dysfunction other than AKI, we should not attribute hypoxic AKI with medullary damage solely to SGLT2i. Yet, it is conceivable that while preserving cortical oxygenation and integrity, SGLT2i may play a permeating role in the development of hypoxic medullary injury, in concert with acute insults such as infections and hemodynamic instability, and with additional predisposing conditions.

## Conclusions

Our study, using serum and urine NGAL and KIM-1, provides insight into AKI in patients treated with SGLT2i. Unaltered KIM-1 in AKI patients might reflect improved cortical oxygenation with SGLT2i, and may explain overall reduced risk of AKI in large clinical studies. By contrast, enhanced expression of sNGAL and especially uNGAL in some patients with AKI provides evidence for medullary tubular injury, likely hypoxic. This may explain occasional reports of AKI in patients on SGLT2i [10,45] that contradict the general safety of these medications in large prospective trials. Definite causative association of ATI and the possible role of SGLT2i currently remain speculative, based on its known impact on medullary oxygenation. However, our findings suggest that if tubular injury develops in patients with AKI while on SGLT2i, it is likely that medullary rather than cortical damage will develop. Further prospective studies including larger numbers of patients are needed to address the independent potential risk to develop tubular injury in patients treated with SGLT2i, perhaps by monitoring changes in urine NGAL excretion along the hospitalization course and comparing the renal outcome in diabetic patients admitted with acute illness while on- or without SGLT2i. Determining the profile of renal biomarkers among hospitalized diabetic patients with AKI not on SGLT2i might further help challenging our hypothesis. The development and use of NGAL kits highly specific to the monomeric biomarker (to exclude detection of NGAL originating from neutrophils) may overcome false positive readings related to infection [43]. For the time being, it seems prudent to state that SGLT2i medications should be transiently suspended in the presence of an acute illness, especially among inpatients, as they might predispose both to pre-renal failure and to hypoxic ATI.
